# Intra-hemispheric integration underlies perception of tilt illusion

**DOI:** 10.1016/j.neuroimage.2018.03.073

**Published:** 2018-07-15

**Authors:** Chen Song, Geraint Rees

**Affiliations:** a Cardiff University Brain Research Imaging Centre, School of Psychology, Cardiff University, Cardiff, UK; b Department of Psychiatry, University of Wisconsin-Madison, Madison, 53719, USA; c Institute of Cognitive Neuroscience, University College London, London, WC1N 3AR, UK; d Wellcome Trust Centre for Neuroimaging, University College London, London, WC1N 3BG, UK

## Abstract

The integration of inputs across the entire visual field into a single conscious experience is fundamental to human visual perception. This integrated nature of visual experience is illustrated by contextual illusions such as the tilt illusion, in which the perceived orientation of a central grating appears tilted away from its physical orientation, due to the modulation by a surrounding grating with a different orientation. Here we investigated the relative contribution of local, intra-hemispheric and global, inter-hemispheric integration mechanisms to perception of the tilt illusion. We used Dynamic Causal Modelling of fMRI signals to estimate effective connectivity in human early visual cortices (V1, V2, V3) during bilateral presentation of a tilt illusion stimulus. Our analysis revealed that neural responses associated with the tilt illusion were modulated by intra- rather than inter-hemispheric connectivity. Crucially, across participants, intra-hemispheric connectivity in V1 correlated with the magnitude of the tilt illusion, while no such correlation was observed for V1 inter-hemispheric connectivity, or V2, V3 connectivity. Moreover, when the illusion stimulus was presented unilaterally rather than bilaterally, the illusion magnitude did not change. Together our findings suggest that perception of the tilt illusion reflects an intra-hemispheric integration mechanism. This is in contrast to the existing literature, which suggests inter-hemispheric modulation of neural activity as early as V1. This discrepancy with our findings may reflect the diversity and complexity of integration mechanisms involved in visual processing and visual perception.

## Highlights

• Involvement of orientation-dependent, intra-hemispheric integration in tilt illusion.
• Correlation between V1 intra-hemispheric connectivity and tilt illusion magnitude.
• No correlation between inter-hemispheric connectivity and tilt illusion magnitude.
• No change in tilt illusion magnitude between bilateral and unilateral presentation.
• Intra-hemispheric connectivity modulates neural responses to bilateral tilt illusion.

## Introduction

Humans can readily and effortlessly perceive a spatially integrated visual world. This integrated nature of visual experience is so fundamental that it often goes unnoticed in day-to-day life, as if our perception can remain unchanged without the capacity of spatial integration. Nevertheless, phenomena such as contextual illusions, in which the perceived features of a stimulus are dissociated from their physical attributes due to the modulation by a spatially adjacent context, explicitly illustrate the integrated nature of visual experience. In fact, spatial integration is implicit in every single visual experience and is the very factor that allows visual experience to be structured (Tononi et al., 2016; Song et al., 2017a). Without spatial integration, visual experience will become fragmented and perception of low-level features such as orientations, distances, sizes, or high-level objects such as faces, scenes, characters, would all cease to be possible.

**Fig. 1:**
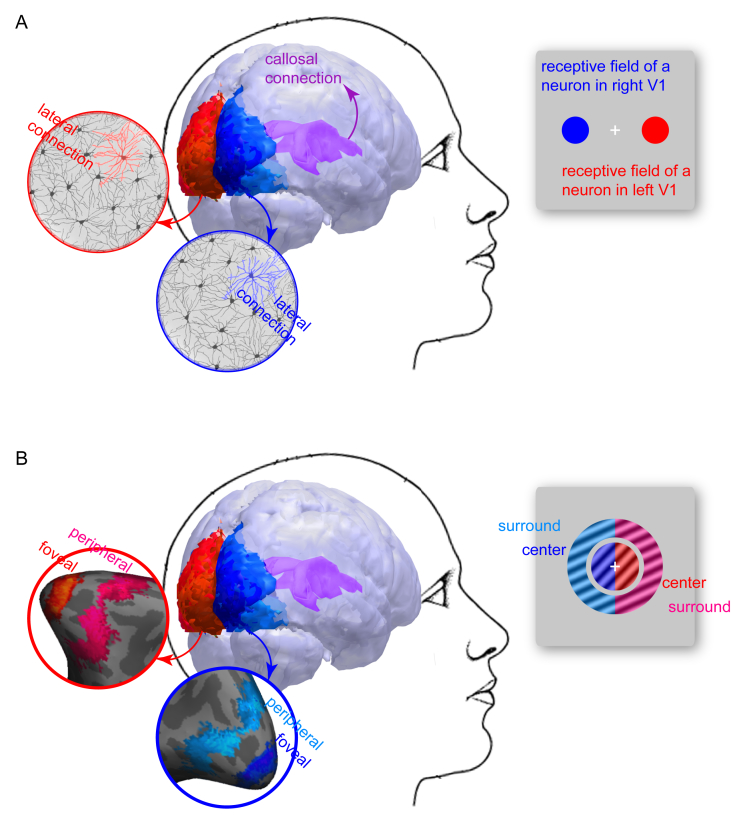
**Neural basis of spatial integration.** (A) Individual neurons in early visual cortices respond only to a limited range of locations in the contralateral hemifield. For example, a neuron in left V1 responds only to specific locations in the right hemifield (red) and a neuron in right V1 to the left hemifield (blue). Despite the spatially localized nature of individual neural response, the lateral connections between adjacent neurons in the same hemisphere (red, blue) and the callosal connections between corresponding neurons in the two hemispheres (purple) allow neurons that respond to different locations in the visual field to communicate with each other, which provides a basis for spatial integration. (B) We investigated the relative contributions of local, intra-hemispheric and global, inter-hemispheric integration to perception of a classical visual contextual illusion, the tilt illusion. In the illusion, the perceived orientation of a central grating appears tilted away from its physical orientation, due to the modulation by a surrounding grating with a different orientation. We measured intra- versus inter-hemispheric connectivity between foveal and peripheral retinotopic regions of the two hemispheres, which responded to the central and surrounding gratings in the two hemifields, respectively (left foveal: red; right foveal: blue; left peripheral: pink; right peripheral: cyan). We then studied how the magnitude of the tilt illusion correlated with intra- versus inter-hemispheric connectivity.

Whereas visual experience is integrated across the entire visual field, individual neurons in visual cortex respond only to a limited range of locations in the visual field (Fig. 1A; Hubel and Wiesel, 1974; Dumoulin and Wandell, 2008; Song et al., 2015). Moreover, the visual cortical system has a contralateral organization, so each hemisphere receives inputs only from the contralateral hemifield (Fig. 1A; Dougherty et al., 2005; Saenz and Fine, 2010; Pietrasanta et al., 2012). The contrast between the integrated nature of visual experience and the spatially localized nature of individual neural responses suggests that it is through the reciprocal connections between spatially tuned neurons that inputs from local parts of the visual field are integrated into a single visual experience (Tononi et al., 1998; Lamme et al., 1998). This integration can happen intra-hemispherically via lateral connections between adjacent neurons (Clarke and Miklossy, 1990; Stettler et al., 2002; Knaap and van der Ham, 2011; Schmidt, 2013; Song et al., 2013a) or inter-hemispherically via callosal connections (Knyazeva et al., 1999, 2005, 2006; Ban et al., 2006; Stark and Milham, 2008).

The lateral and callosal connections provide bases for intra- and inter-hemispheric integration. However, the contributions of different integration mechanisms to visual perception remain unclear. On the one hand, cases of split-brain patients, for whom the loss or surgical removal of callosal connections does not impair their ability to have visual experience within individual hemifields, indicate that inter-hemispheric integration may not be as essential to visual perception as intra-hemispheric integration (Zaidel and Iacoboni, 2003). It is plausible that in healthy participants, intra-hemispheric integration alone can shape perception of bilateral stimuli. On the other hand, several fMRI and EEG studies show that neural activity as early as primary visual cortex (V1) is modulated by inputs from both contralateral and ipsilateral hemifields (Knyazeva et al., 1999, 2005, 2006; Ban et al., 2006; Stark and Milham, 2008). For example, interhemispheric EEG coherence is enhanced by a single grating stimulus that extends across the vertical meridian (Knyazeva et al., 1999). Moreover, retinotopic responses to a unilateral stimulus are enhanced when another stimulus is presented at the point-symmetrical position of the opposite hemifield (Ban et al., 2006). Whereas these studies hint towards a joint contribution of intra- and inter-hemispheric integration to visual perception, they did not measure any perceptual effects but measured neural activity only. Since not all changes in neural activity will necessarily contribute to perception (Rees et al., 2002; Tononi et al., 2016; Song and Yao, 2016), it is difficult to infer the behavioral significances of these findings.

Here we compared the relative contributions of local, intra-hemispheric and global, inter-hemispheric integration to perception of a classical visual contextual illusion, the tilt illusion. In the illusion, the perceived orientation of a central grating is tilted away from its physical orientation, due to the modulation by a surrounding grating with a different orientation (Schwartz et al., 2007; Clifford, 2014). The magnitude of the tilt illusion depends on the spatial proximity between the central and surrounding gratings, suggesting the likely involvement of local, intra-hemispheric integration mechanisms (Wenderoth and Johnstone, 1988). To test whether perception of the tilt illusion involves both intra- and inter-hemispheric integration, or mainly intra-hemispheric integration, we studied how the illusion magnitude correlated with intra-hemispheric versus inter-hemispheric connectivity between foveal and peripheral retinotopic regions that responded to the central and surrounding gratings, respectively (Fig. 1B). We used Dynamic Casual Modelling of fMRI signals to disentangle intra- versus inter-hemispheric connectivity in early visual cortices, and investigated how inter-individual variability in the magnitude of the tilt illusion correlated with individual differences in intra-, inter-hemispheric connectivity. This individual difference approach created variability in the illusion magnitude without changing the illusion stimulus and allowed dissociation across participants between the perceptual and the stimulation effects.

## Materials and methods

In the first set of experiments (fMRI, psychophysics), we tested whether the magnitude of the tilt illusion measured under bilateral presentation of illusion stimulus correlated with intra- or inter-hemispheric connectivity. We used DCM analysis of fMRI signals (Friston, 2011) to disentangle intra- versus inter-hemispheric connectivity between foveal and peripheral retinotopic regions of the same versus opposite hemisphere. To anticipate our findings, the magnitude of the tilt illusion correlated with intra- but not with inter-hemispheric connectivity. In the second set of experiments (psychophysics), we further tested how the magnitude of the tilt illusion changed with stimulus configurations (size, orientation) and stimulus presentations (bilateral, unilateral).

### Participants and apparatus

Twenty healthy volunteers (ten females, aged 19–34), with normal or corrected-to-normal vision and no history of neurological or psychiatric illness, gave written informed consent to participate in this study which was approved by the UCL Research Ethics Committee. FMRI experiments took place in a Siemens Trio 3T MRI scanner with a 32-channel head coil. Structural MRI data were acquired using a T1-weighted MDEFT sequence (resolution: 1 mm isotropic; slice number: 176; matrix size: 256 × 240; TR: 7.92 ms; TE: 2.48 ms). Functional MRI data were acquired using a 3D EPI sequence (resolution: 1.5 mm isotropic; slice number: 40; matrix size: 128 × 128; volume TR: 3.2 s; TE: 32.86 ms) and preprocessed in SPM8 (http://www.fil.ion.ucl.ac.uk/spm) using intensity bias correction, realignment, unwarping, coregistration with structural MRI and physiological noise correction. Psychophysics experiments took place in a dark room with the monitor providing the only source of light.

### FMRI experiments

#### Stimulus

**Fig. 2:**
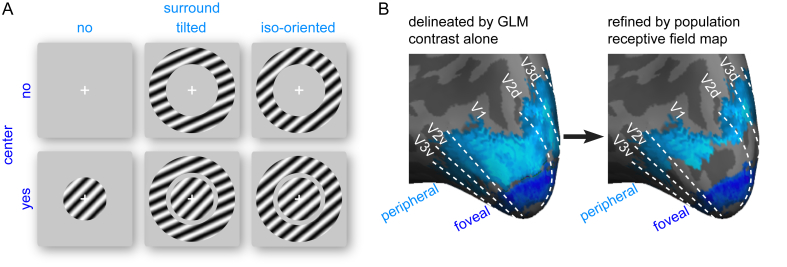
**Foveal and peripheral retinotopic regions.** (A) FMRI data were acquired when participants viewed a blank screen, a central grating alone, a surrounding grating alone, or a central and a surrounding grating together, in different experimental blocks. Two types of surrounding grating were used, one tilted relative to the central grating and one iso-oriented to the central grating. (B) Foveal (blue) and peripheral (cyan) retinotopic regions were first delineated by General Linear Model (GLM) analysis contrasting blocks of central and surrounding gratings (left panel), and then refined by excluding voxels whose population receptive fields overlapped with the surrounding and central gratings, respectively (right panel). The % of voxels excluded in the refinement was 7.86 ± 0.40% for foveal retinotopic regions and 41.71 ± 0.91% for peripheral retinotopic regions.

Block-design fMRI data were acquired in four experimental runs (180 acquisition volumes per run). In each run, six different stimuli (Fig. 2A), a blank screen, a 45° central grating, a 45° surrounding grating, a 60° surrounding grating, a 45° central grating with a 45° surrounding grating, a 45° central grating with a 60° surrounding grating, were presented for a total of six blocks per stimulus, five acquisition volumes per block and counterbalanced block sequence across runs. The orientation of the central grating (45°) and the orientation difference between the central and surrounding gratings (15°) were chosen to maximize the illusion magnitude. Specifically, existing psychophysical evidence suggested a maximal illusion at a 15° orientation difference between the central and surrounding gratings (Wenderoth and Johnstone, 1988; Clifford et al., 2000). Moreover, because of the oblique effect, the tilt illusion is the strongest when the central grating is oblique (45°) as opposed to vertical or horizontal (Clifford, 2014).

Visual stimuli were projected on a screen (28.6 × 21.5 cm) in the back of the scanner and viewed through a head-coil mirror at a distance of 85 cm. The central and surrounding gratings (spatial frequency: 2 cycles per degree; contrast: 100%) covered 0.25–1.75 and 2.4–7.2 degrees of visual field eccentricity, respectively. The sizes of the central and surrounding gratings were chosen according to the scaling law of cortical magnification factor (Sereno et al., 1995), to ensure equal size of foveal and peripheral retinotopic regions. The eccentricity gap between the central and surrounding gratings was chosen according to the population receptive field size (Dumoulin and Wandell, 2008), to allow for a reliable separation between foveal and peripheral retinotopic regions. The eccentricity gap within the central grating helped to avoid the activation of foveal confluence where the boundaries between early visual cortices were difficult to delineate.

#### Task

Throughout the experiment, participants passively viewed the orientation of grating stimuli, while maintaining central fixation by detecting the color changes of the fixation cross with a button press. This passive viewing paradigm helped to minimize the confounding factors, such as allocation of attention and its inter-individual difference. Yet, to account for the potential influences of experimental task, we further conducted a control experiment, in which participants actively detected the intermittent orientation changes of the central grating (frequency of change: 0–2 per block; degree of change: 1.5 times the orientation discrimination threshold), while maintaining central fixation and being monitored by an eye tracker (Eyelink 1000). This active viewing paradigm matched the tasks in the psychophysical experiments. All participants took part in the passive viewing experiment and ten participants took part in the active viewing experiment. The measure of effective connectivity was consistent between the two paradigms. Reported in the results were the passive viewing data.

#### Retinotopy

In addition to the main experiments, all participants underwent phase-encoded retinotopic mapping, viewing full-contrast flickering checkerboard wedges (radius: 7.2 degrees of visual field eccentricity; width: 40°) rotating smoothly around a fixation cross for ten cycles at a speed of twenty volumes per cycle (Sereno et al., 1995). They were instructed to detect a small pattern shift of the checkboard wedges (frequency of change: 1 per cycle; duration of change: 200 ms), while maintaining central fixation and being monitored by an eye tracker (Eyelink 1000). Fast Fourier transform was applied to BOLD time series to extract the phase and power at the stimulation frequency. The resulting phase maps was displayed on cortical surfaces reconstructed in Freesurfer (http://surfer.nmr.mgh.harvard.edu), to delineate the boundaries between V1, V2, V3 by mirror reversals in the phase maps.

All participants also underwent population-receptive-field mapping, viewing full-contrast flickering checkerboard bars (width: 1.8 degrees of visual angle) moving smoothly in the visual field for eight cycles at a speed of sixteen volumes per cycle (Dumoulin and Wandell, 2008). The bars were oriented at one of the four orientations (0°, 45°, 90°, 135°) and moved along the corresponding orthogonal direction (north or south for 0° bar, northwest or southeast for 45° bar, west or east for 90° bar, northeast or southwest for 135° bar). A blank screen was inserted into the last quarter of the second, fourth, sixth and eighth cycles to provide a baseline condition that improved the accuracy of the measures. Participants passively viewed the stimuli while maintaining central fixation by detecting the color changes of fixation cross with a button press. BOLD time series of each voxel in V1, V2, or V3 were fitted with a two-dimensional Gaussian function multiplied by the stimulus location function and convolved with hemodynamic response function. The two-dimensional Gaussian function characterized the visual field range that the voxel responded to, namely the population receptive field (Dumoulin and Wandell, 2008).

#### Region-of-interest (ROI)

Foveal and peripheral reinotopic regions were delineated by General Linear Model (GLM) analysis contrasting blocks of central and surrounding gratings (collapsing across conditions), at a threshold of p < 0.001 (uncorrected). To avoid spillover of signals between adjacent ROIs, the foveal and peripheral retinotopic regions delineated in this way were further refined (Fig. 2B), by excluding voxels whose population receptive fields overlapped with the surrounding and central gratings, respectively. Based on the boundaries of early visual cortices measured from phase-encoded retinotopic mapping and the delineation of foveal and peripheral regions, twelve ROIs, left foveal V1, right foveal V1, left peripheral V1, right peripheral V1, left foveal V2, right foveal V2, left peripheral V2, right peripheral V2, left foveal V3, right foveal V3, left peripheral V3 and right peripheral V3 were defined. The sizes of ROIs (before refinement) were 2706.7 ± 86.2 mm^3^ for foveal V1, 2519.9 ± 64.2 mm^3^ for peripheral V1, 2517.7 ± 79.2 mm^3^ for foveal V2, 2502.0 ± 77.4 mm^3^ for peripheral V2, 1997.2 ± 89.4 mm^3^ for foveal V3, 2292.0 ± 82.1 mm^3^ for peripheral V3. The % of voxels excluded in the refinement was 5.57 ± 0.44% for foveal V1, 41.70 ± 1.49% for peripheral V1, 7.95 ± 0.55% for foveal V2, 39.15 ± 1.60% for peripheral V2, 10.07 ± 0.72% for foveal V3 and 44.30 ± 1.55% for peripheral V3.

#### Effective connectivity

Dynamic Causal Modelling (DCM) analysis was used to estimate effective connectivity, within and across hemispheres, between foveal and peripheral retinotopic regions of V1, V2 and V3. Functional connectivity analysis measures only the statistical dependence (correlation coefficient) between BOLD time series of different ROIs and cannot distinguish whether such statistical dependence arises from the connectivity between these ROIs, or from shared and/or changed inputs to these ROIs (Friston, 2011). By contrast, the DCM effective connectivity analysis we used can disambiguate the effects of confounding factors from the effects of underlying connectivity (Friston, 2011), through Bayesian comparisons between different models, each incorporating a connectivity and input structure that is hypothesized to explain the empirically observed BOLD time series. DCM analysis is therefore particularly suited for estimating the changes in connectivity across different visual inputs, such as from non-contextual (central grating alone, surrounding grating alone, blank screen) to contextual stimulation (central and surrounding gratings together).

To infer neural connectivity from BOLD signals, DCM analysis applies hemodynamic state equations to model the neurovascular coupling between BOLD signals and neural activities, and neural state equations to model how neural activities are shaped by the connectivity between neural populations and the inputs to neural populations (Friston et al., 2000; Deco et al., 2008). Different models incorporating different hypothesis of connectivity and input structure are constructed, and Bayesian comparisons are used to identify the model with the highest posterior probability (Penny et al., 2006). The posterior probability of a model is a function of its prior probability and its model evidence. The prior probability reflects prior knowledge about the anatomical plausibility of the model structure. When there exists no strong prior against or for specific models, different models are given the same prior probability. The model evidence quantifies the probability of the observed data given the model. It is a reflection of both how accurate the model fits the data (the more accurate, the higher the model evidence) and how simple the model structure is (the simpler, the higher the model evidence). Free energy is used in DCM analysis as an approximation for model evidence (Penny, 2012).

**Fig. 3:**
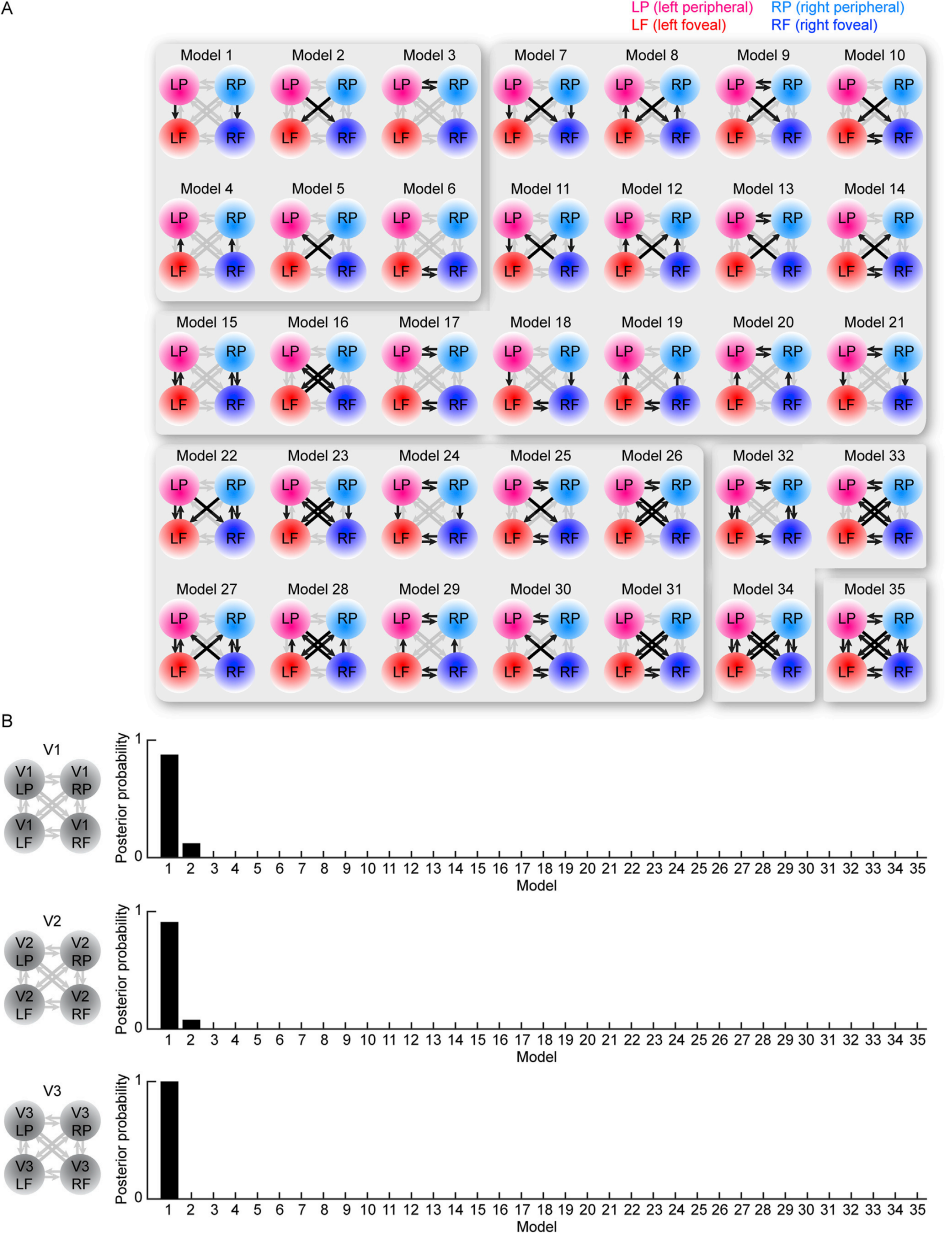
**Dynamic Causal Modelling analysis.** (A) To estimate intra- versus inter-hemispheric connectivity, thirty-five anatomically plausible DCM models comprising four inter-connected retinotopic regions, left foveal (red), right foveal (blue), left peripheral (pink), right peripheral (cyan), were constructed respectively in V1, V2 and V3. Each model incorporated a prior hypothesis of the changes in intra- and/or inter-hemispheric effective connectivity between these retinotopic regions from non-contextual (central grating alone, surrounding grating alone, blank screen) to contextual stimulation (central and surrounding gratings together). Models one to six hypothesized changes in one specific type of connectivity (model one: intra-hemispheric from peripheral to foveal; model two: inter-hemispheric from peripheral to foveal; model three: inter-hemispheric from peripheral to peripheral; model four: intra-hemispheric from foveal to peripheral; model five: inter-hemispheric from foveal to peripheral; model six: inter-hemispheric from foveal to foveal). The remaining models hypothesized a combination of two (models seven to twenty-one), three (models twenty-two to thirty-one), four (models thirty-two to thirty-four), or all (model thirty-five) of these changes. The connectivities under hypothetical changes are represented by black arrows and the rest by grey arrows. (B) Bayesian comparisons were used to identify the model with the highest posterior probability, separately for V1, V2 and V3. The model which hypothesized changes in intra-hemispheric connectivity from peripheral to foveal retinotopic regions (model one) had the highest posterior probability. The remaining models, hypothesizing changes in intra-hemispheric connectivity from foveal to peripheral retinotopic regions, or inter-hemispheric connectivity, or their combinations, all had very low posterior probability.

For each participant, thirty-five anatomically plausible DCM models comprising four inter-connected retinotopic regions, left foveal, right foveal, left peripheral, right peripheral, were constructed respectively in V1, V2 and V3 (Fig. 3A). Each model incorporated a prior hypothesis of the changes in intra- and/or inter-hemispheric effective connectivity between these retinotopic regions from non-contextual (central grating alone, surrounding grating alone, blank screen) to contextual stimulation (central and surrounding gratings together). Specifically, model one hypothesized changes in intra-hemispheric connectivity from peripheral to foveal retinotopic regions, model two inter-hemispheric connectivity from peripheral to foveal retinotopic regions, model three inter-hemispheric connectivity between peripheral retinotopic regions, model four intra-hemispheric connectivity from foveal to peripheral retinotopic regions, model five inter-hemispheric connectivity from foveal to peripheral retinotopic regions, and model six inter-hemispheric connectivity between foveal retinotopic regions. The remaining models hypothesized a combination of two (models seven to twenty-one), three (models twenty-two to thirty-one), four (models thirty-two to thirty-four), or all (model thirty-five) of these changes. All models had the same prior probability and Bayesian comparisons were used to identify the model with the highest posterior probability (Fig. 3B).

As DCM analysis is limited by the number of ROIs that it can include in a single model, we confined our current DCM models to testing intra- versus inter-hemispheric connectivity within the same visual area and did not include changes in effective connectivity between different visual areas. In our previous study where we tested the involvement of intra-areal versus inter-areal connectivity in perception of the tilt illusion, we found that the magnitude of the tilt illusion correlated with intra-areal connectivity from peripheral to foveal V1, but not with feedforward or feedback connectivity between V1, V2 and V3 (Song et al., 2013a). However, that study did not disentangle intra- versus inter-hemispheric connectivity, and the results could reflect either a combination of intra- and inter-hemispheric contributions, or an intra-hemispheric contribution alone. In the current study, we asked a different question: whether perception of the tilt illusion involves both intra- and inter-hemispheric integration, or mainly intra-hemispheric integration. Since our previous study already showed no involvement of inter-areal connectivity in perception of the tilt illusion, our current DCM models did not include changes in effective connectivity between different visual areas.

### Psychophysics experiments

#### Stimulus

The magnitude of the tilt illusion was measured on a per-participant basis for five different stimulus configurations in five separate experiments. All participants took part in the first three experiments. Fifteen participants took part in all five experiments. Visual stimuli were presented on a 22-inch CRT monitor (41 × 30.6 cm) and viewed through a chin and forehead rest at a distance of 67 cm.

In the first experiment, we used stimulus configuration identical to the fMRI experiments, in which the central and surrounding gratings (spatial frequency: 2 cycles per degree; contrast: 70%) were oriented at 45° and 60° respectively, and covered 0.25–1.75 and 2.4–7.2 degrees of visual field eccentricity respectively. This stimulus configuration was optimal for fMRI, where the large stimulus size contributed to the widespread coverage of visual field eccentricity, the eccentricity gap between the central and surrounding gratings contributed to the reliable separation of foveal versus peripheral retinotopic regions, and the eccentricity gap within the central grating helped to avoid the activation of the foveal confluence.

In the second and third experiments, we used a slightly different stimulus configuration optimal for psychophysics, in which the central and surrounding gratings (spatial frequency: 2 cycles per degree; contrast: 70%) were oriented at 45° and 60° respectively (second experiment), or 0° and 15° respectively (third experiment), and covered 0–0.75 and 0.75–3 degrees of visual field eccentricity respectively. We found that the illusion magnitude exhibited inter-individual correlation across stimulus size (r = 0.69 [0.41, 0.86], p < 0.001, N = 20) and orientation (r = 0.87 [0.73, 0.95], p < 10^−6^, N = 20), suggesting that the magnitude of the tilt illusion reflected a stimulus-independent trait.

**Fig. 4:**
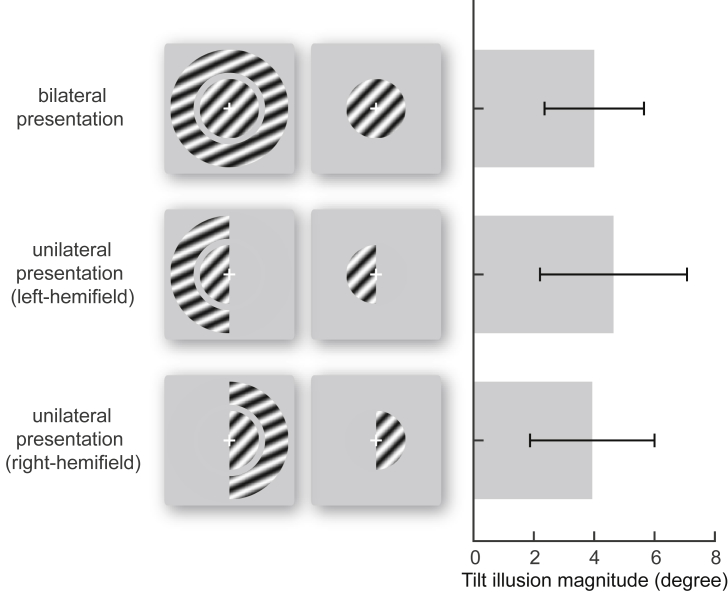
**Similar illusion magnitude comparing bilateral and unilateral tilt illusion.** We compared the magnitude of the tilt illusion measured under different presentations of illusion stimulus, where the central and surrounding gratings spanned both hemifields or were confined to left or right hemifield. The comparisons showed that, when the illusion stimulus changed from bilateral to unilateral presentation, the illusion magnitude did not change significantly. Bar charts reflect mean and 95% C.I. across participants.

In the fourth and fifth experiments, we kept stimulus configuration optimal for psychophysics, while changing stimulus presentation from bilateral to unilateral (Fig. 4). Specifically, the central and surrounding gratings (spatial frequency: 2 cycles per degree; contrast: 70%) were oriented at 45° and 60° respectively, and covered 0–0.75 and 0.75–3 degrees of visual field eccentricity in left (fourth experiment) or right (fifth experiment) visual field respectively. We found that the illusion magnitude measured under bilateral presentation correlated with that measured under unilateral left-hemifield (r = 0.84 [0.70, 0.95], p < 0.0001, N = 15) or right-hemifield presentation (r = 0.77 [0.68, 0.95], p < 0.001, N = 15).

#### Task

To minimize the confounding influences from variables of no interest, the same psychophysical procedures were used in all five experiments. Participants first completed a match-to-standard session in which a testing central grating (one without surround) and a reference central grating (one surrounded by an annular grating) were presented intermittently (500 ms each, separated by a 500 ms interval of blank screen), and participants manually adjusted the orientation of the testing central grating till it matched the perceived orientation of the reference central grating. Participants then completed a staircase session, which measured orientation discrimination threshold through standard 2-up-1-down staircase procedures. The point of subjective equality measured from the match-to-standard session and the orientation discrimination threshold measured from the staircase session were used to guide the choices of stimulus parameters in the subsequent two-alternative-forced choice session.

A single trial in the two-alternative-forced choice session comprised the successive presentation of two central gratings, a reference one surrounded by an annular grating and a testing one with no surroundings. The orientation of the reference grating was kept constant. The orientation of the testing grating was varied between seven values (sixteen trials per value) around the point of subjective equality (measured from the match-to-standard session), with the step size equal to the orientation discrimination threshold (measured from the staircase session). The two central gratings were presented in a randomized order for 300 ms each, separated by a 500 ms interval of blank screen. After they disappeared, participants made an unspeeded forced-choice response as to whether the central grating in the second presentation, compared with the one in the first presentation, was rotated clockwise or counterclockwise.

#### Illusion magnitude

Data from the two-alternative-forced choice session were fitted with a psychometric function to measure the 50% threshold point at which the two central gratings appeared perceptually equal in orientation despite their physical difference in orientation (point of subjective equality). The magnitude of the tilt illusion was quantified as the physical orientation difference between the two central gratings at the point of subjective equality.

## Results

### Intra-hemispheric connectivity modulates neural responses to bilateral tilt illusion

**Fig. 5:**
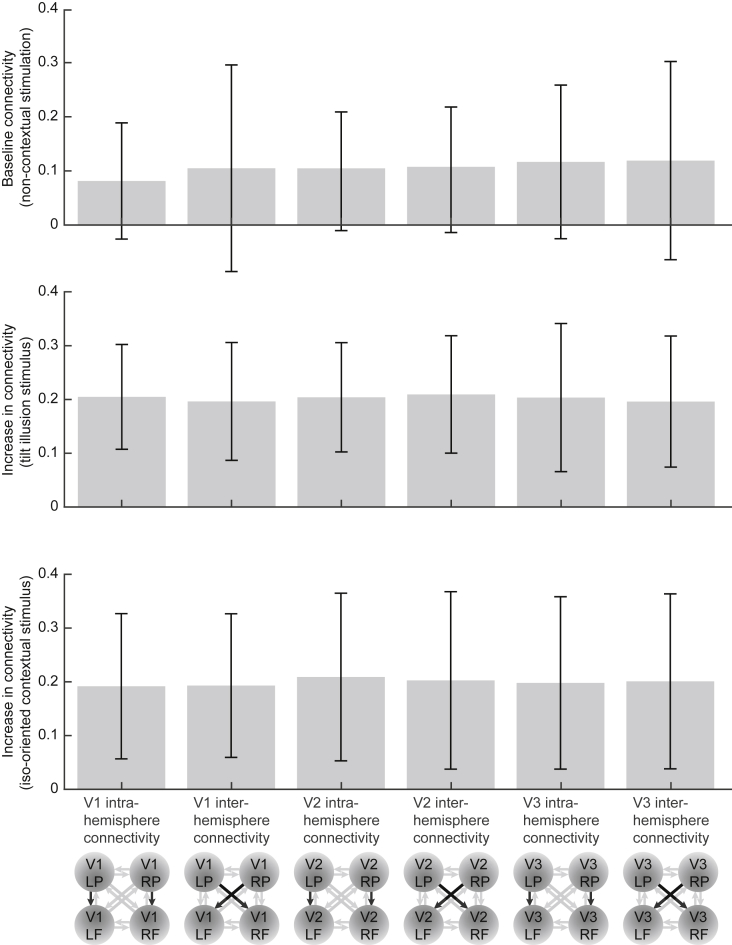
**Intra- and inter-hemispheric connectivity.** Under baseline condition (central grating alone, surrounding grating alone, blank screen), intra- and inter-hemispheric effective connectivity from peripheral to foveal retinotopic regions was very weak and not significantly different from zero. Under contextual stimulation (central and surrounding gratings together), significant increases in effective connectivity were observed, regardless of whether the surrounding grating had a different orientation to the central grating (tilt illusion stimulus) or an identical orientation to the central grating (iso-oriented contextual stimulus). The common increases in effective connectivity from non-contextual to contextual stimulation, observed in all early visual cortices (V1, V2, V3) for both tilt illusion stimulus and iso-oriented contextual stimulus, suggest a generic, orientation-independent modulation exerted by contextual stimulation. Bar charts reflect mean and 95% C.I. of the maximum a posteriori (MAP) estimates across participants.

Dynamic Causal Modelling (DCM) analysis was used to estimate effective connectivity, within and across hemispheres, between foveal and peripheral retinotopic regions of V1, V2 and V3. Under non-contextual stimulation (central grating alone, surrounding grating alone, blank screen), effective connectivity from peripheral to foveal retinotopic regions was very weak and not significantly different from zero (Fig. 5; mean and 95% C.I. of maximum a posteriori estimates = 0.081 [-0.027, 0.189] for V1 intra-hemispheric, 0.105 [-0.087, 0.297] for V1 inter-hemispheric, 0.105 [-0.006, 0.215] for V2 intra-hemispheric, 0.107 [-0.010, 0.224] for V2 inter-hemispheric, 0.117 [-0.026, 0.259] for V3 intra-hemispheric, 0.119 [-0.066, 0.303] for V3 inter-hemispheric). By contrast, under contextual stimulation (central and surrounding gratings together), significant increases in effective connectivity were observed (Fig. 5).

The increases in effective connectivity were observed for both tilt illusion stimulus and iso-oriented contextual stimulus, where the surrounding grating had a different and an identical orientation to the central grating, respectively. Moreover, the degree of increase was comparable between tilt illusion stimulus and iso-oriented contextual stimulus, as well as between intra- and inter-hemispheric connectivity. Specifically, under tilt illusion stimulus, the increases were 0.205 [0.108, 0.303] for V1 intra-hemispheric, 0.197 [0.087, 0.306] for V1 inter-hemispheric, 0.204 [0.103, 0.306] for V2 intra-hemispheric, 0.210 [0.100, 0.319] for V2 inter-hemispheric, 0.204 [0.066, 0.342] for V3 intra-hemispheric, and 0.196 [0.074, 0.318] for V3 inter-hemispheric effective connectivity. Under iso-oriented contextual stimulus, the increases were 0.192 [0.057, 0.327] for V1 intra-hemispheric, 0.193 [0.059, 0.327] for V1 inter-hemispheric, 0.209 [0.053, 0.365] for V2 intra-hemispheric, 0.203 [0.038, 0.368] for V2 inter-hemispheric, 0.198 [0.038, 0.358] for V3 intra-hemispheric, and 0.201 [0.038, 0.364] for V3 inter-hemispheric connectivity.

To infer whether intra- or inter-hemispheric connectivity modulated neural responses to the tilt illusion, thirty-five different DCM models, each incorporating a prior hypothesis of the changes in intra- and/or inter-hemispheric effective connectivity from non-contextual to contextual stimulation, were constructed respectively in V1, V2 and V3 (Fig. 3A). Bayesian comparisons were used to identify the model with the highest posterior probability (Fig. 3B). All models were given the same prior probability. The model that hypothesized changes in intra-hemispheric connectivity from peripheral to foveal V1 (model one) had the highest posterior probability; the remaining models, hypothesizing changes in inter-hemispheric connectivity from peripheral to foveal V1 (model two), inter-hemispheric connectivity between peripheral V1 of the two hemispheres (model three), intra-hemispheric connectivity from foveal to peripheral V1 (model four), inter-hemispheric connectivity from foveal to peripheral V1 (model five), inter-hemispheric connectivity between foveal V1 of the two hemispheres (model six), or any of their combinations (models seven to thirty-five), all had very low posterior probability. The same results were observed for V2 and V3. These results suggest that neural responses associated with the tilt illusion are modulated by intra- rather than inter-hemispheric connectivity from peripheral to foveal retinotopic regions.

### Intra-hemispheric connectivity correlates with the magnitude of bilateral tilt illusion

**Fig. 6:**
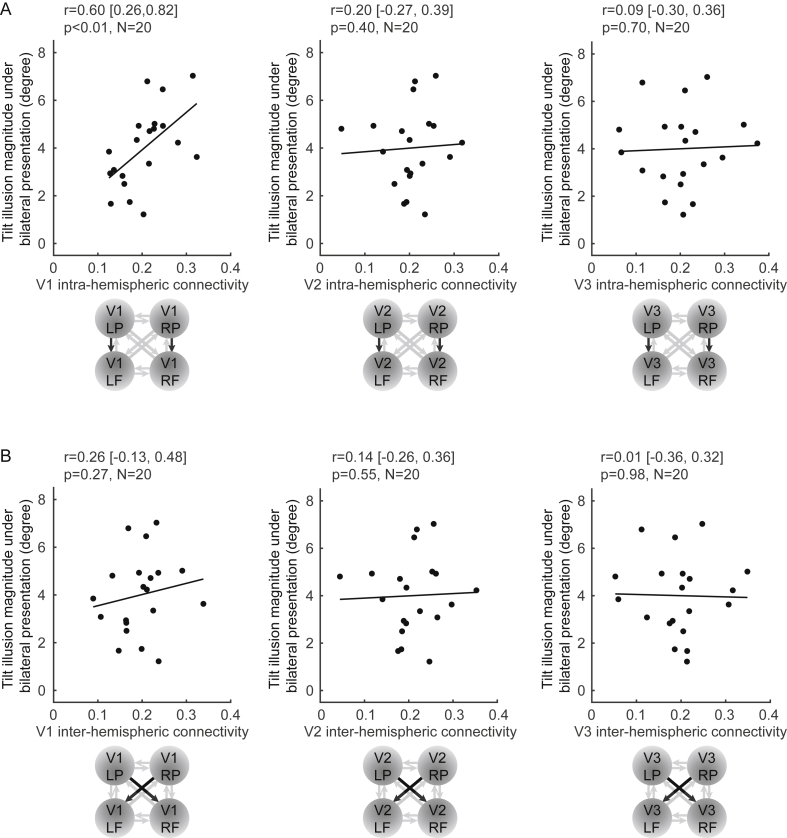
**Intra-hemispheric connectivity correlates with the magnitude of bilateral tilt illusion.** The magnitude of the tilt illusion (measured under bilateral presentation of illusion stimulus) was plotted against intra-, inter-hemispheric connectivity (averaged between two hemispheres). A significant correlation with the illusion magnitude was observed for intra-hemispheric connectivity from peripheral to foveal V1, but not for V1 inter-hemispheric connectivity, V2 intra-hemispheric connectivity, V2 inter-hemispheric connectivity, V3 intra-hemispheric connectivity, or V3 inter-hemispheric connectivity. Each point represents a participant. Statistical values reflect Spearman's rho and 95% bootstrap C.I. after correction for multi-comparisons using the false discovery rate (FDR).

We then studied how the effective connectivity measured from fMRI experiments correlated with the magnitude of the tilt illusion measured in independent psychophysics experiments. We found that across participants, the illusion magnitude correlated significantly with intra-hemispheric connectivity from peripheral to foveal V1 (Fig. 6A; r = 0.60 [0.26, 0.82], p < 0.01, N = 20). By contrast, while V1 intra- and inter-hemispheric effective connectivity were similar in value, there was no significant correlation between the magnitude of the tilt illusion and inter-hemispheric connectivity from peripheral to foveal V1 (Fig. 6B; r = 0.26 [-0.13, 0.48], p = 0.27, N = 20).

We also found that the correlation with the illusion magnitude was specific to effective connectivity measured under tilt illusion stimulus and not observed for effective connectivity measured under iso-oriented contextual stimulus (r = 0.22 [-0.11, 0.71], p = 0.35, N = 20). Furthermore, the correlation was specific to V1 and not observed for V2 intra-hemispheric connectivity (Fig. 6A; r = 0.20 [-0.27, 0.39], p = 0.40, N = 20), V2 inter-hemispheric connectivity (Fig. 6B; r = 0.14 [-0.26, 0.36], p = 0.55, N = 20), V3 intra-hemispheric connectivity (Fig. 6A; r = 0.09 [-0.30, 0.36], p = 0.70, N = 20), or V3 inter-hemispheric connectivity (Fig. 6B; r = 0.01 [-0.36, 0.32], p = 0.98, N = 20).

### Similar illusion magnitude comparing bilateral and unilateral tilt illusion

These results, based on bilateral presentation of illusion stimulus, suggest that perception of the tilt illusion involves mainly intra-hemispheric integration. Consistent with these results, we found that when the illusion stimulus changed from bilateral to unilateral presentation, the illusion magnitude did not change significantly (Fig. 4; bilateral to left-hemifield: T (14) = 1.35, p = 0.20, N = 15; bilateral to right-hemifield: T (14) = 0.62, p = 0.54, N = 15).

**Fig. 7:**
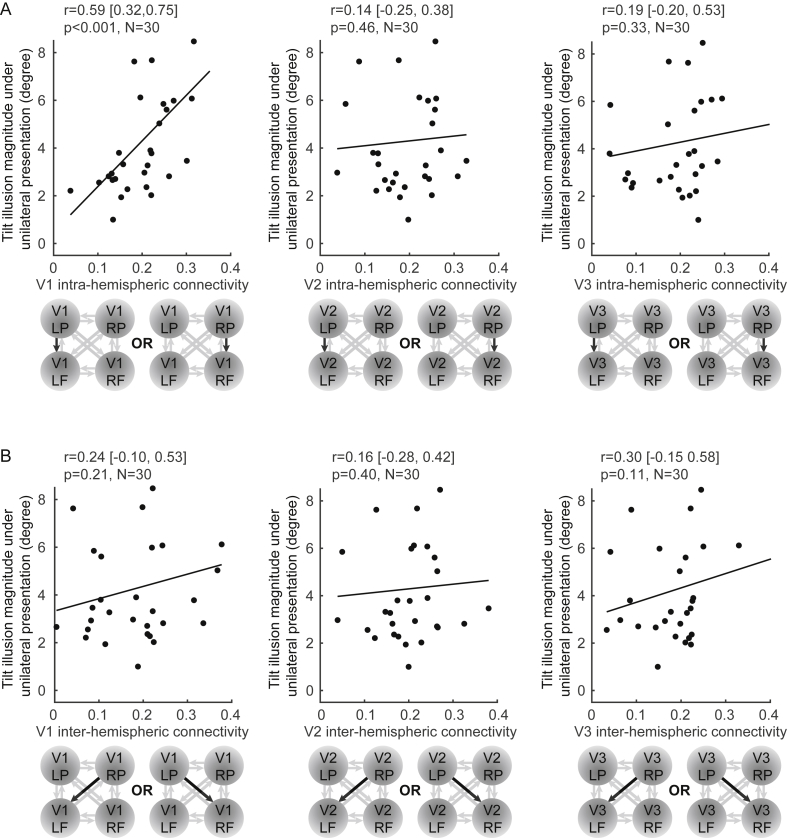
**Intra-hemispheric connectivity correlates with the magnitude of unilateral tilt illusion.** The magnitude of the tilt illusion (measured under unilateral presentation of illusion stimulus) was plotted against intra-, inter-hemispheric connectivity (taken separately for two hemispheres). A significant correlation with the illusion magnitude was observed for intra-hemispheric connectivity from peripheral to foveal V1, but not for V1 inter-hemispheric connectivity, V2 intra-hemispheric connectivity, V2 inter-hemispheric connectivity, V3 intra-hemispheric connectivity, or V3 inter-hemispheric connectivity. Each point represents a participant. Statistical values reflect Spearman's rho and 95% bootstrap C.I. after correction for multi-comparisons using the false discovery rate (FDR).

Moreover, we found that the illusion magnitude measured under unilateral presentation of illusion stimulus correlated significantly with V1 intra-hemispheric connectivity (Fig. 7A; r = 0.59 [0.32, 0.75], p < 0.001, N = 30), but not with V1 inter-hemispheric connectivity (Fig. 7B; r = −0.24 [-0.10, 0.53], p = 0.21, N = 30), V2 intra-hemispheric connectivity (Fig. 7A; r = 0.14 [-0.25, 0.38], p = 0.46, N = 30), V2 inter-hemispheric connectivity (Fig. 7B; r = 0.16 [-0.28, 0.42], p = 0.40, N = 30), V3 intra-hemispheric connectivity (Fig. 7A; r = 0.19 [-0.20, 0.53], p = 0.33, N = 30), or V3 inter-hemispheric connectivity (Fig. 7B; r = 0.30 [-0.15, 0.58], p = 0.11, N = 30). These results provided further support for an intra-hemispheric integration mechanism underlying the tilt illusion.

## Discussion

Spatial integration is the basis of human visual perception, but it is often taken for granted. Many studies focus on feature or object perception that is derived from the spatial structure of visual experience, without first addressing the neural basis of spatial integration. To support spatial integration, neural responses need to have both the capacity to differentiate and the capacity to integrate across individual locations in the visual field (Tononi et al., 2016; Song et al., 2017a). If different locations in the visual field do not have distinct neural representations, then from a purely neuronal perspective, these locations are not different from one another and are effectively just one location. The capacity of differentiation is achieved through receptive field, where visual cortical neurons respond selectively to limited locations in the visual field, rather than uniformly to any location in the visual field (Fig. 1A). However, differentiation alone is not enough. Neural representations of different locations need to be not just distinct but also integrable. The capacity of integration is achieved through neural connections, where visual cortical neurons responding to adjacent locations are connected intra-hemispherically by lateral connections and inter-hemispherically by callosal connections (Fig. 1A).

Despite these theoretical foundations, empirical evidence that links perceptual integration to intra-hemispheric lateral connections or inter-hemispheric callosal connections remains sparse. Here we used fMRI DCM analysis in combination with psychophysics to address the neural basis of perceptual integration in a classical visual contextual illusion, the tilt illusion. In the first set of experiments (fMRI, psychophysics), we used bilateral presentation of illusion stimulus, to compare the contributions of local, intra-hemispheric and global, inter-hemispheric integration to perception of the tilt illusion. We found that across participants, intra-hemispheric connectivity in V1 correlated with the magnitude of the tilt illusion, while no such correlation was observed for V1 inter-hemispheric connectivity, V2 intra-hemispheric connectivity, V2 inter-hemispheric connectivity, V3 intra-hemispheric connectivity, or V3 inter-hemispheric connectivity. Moreover, the correlation was specific to V1 intra-hemispheric connectivity measured during perception of the tilt illusion and not observed for V1 intra-hemispheric connectivity measured during perception of a control, iso-oriented contextual stimulus. In the second set of experiments (psychophysics), we studied how the magnitude of the tilt illusion changed with stimulus presentations. We found that when the illusion stimulus was presented bilaterally rather than unilaterally, the illusion magnitude did not change.

Together our findings suggest that perception of the tilt illusion reflects an intra-hemispheric integration mechanism. Moreover, the selective correlation between the magnitude of the tilt illusion and intra-hemispheric connectivity in V1 (as opposed to V2, V3) measured during perception of the tilt illusion (as opposed to during perception of the iso-oriented contextual stimulus) suggests the involvement of an orientation-dependent integration mechanism. In early visual cortices, neurons exhibit clear orientation preferences such that their response is the strongest for a preferred orientation and decays as the stimulus orientation deviates from their preferred orientation (Ringach et al., 2002). Crucially, the orientation preference has a topographical representation in which intra-areal connections preferentially link neurons with similar orientation preference (Bosking et al., 1997; Bock and Reid, 2011; Li et al., 2012). The topographical representation of stimulus orientation is particularly prominent in V1, while in V2 or V3, the representation of stimulus orientation is interleaved with the representation of stimulus color (according to intrinsic-signal optical imaging data in non-human primates; Chklovskii and Koulakov, 2004; Kaskan et al., 2008).

As a result of this V1 representation of stimulus orientation, neural responses to the surrounding grating can modulate and repulsively shift neural responses to the central grating, through intra-areal connections from peripheral to foveal V1; this repulsive shift can then give rise to a corresponding shift in the perceived orientation of central grating, and to the phenomenon of the tilt illusion (Schwartz et al., 2009). Depending on the exact orientations of surrounding and central gratings, different neural populations in V1 will be activated. The selective correlation between the magnitude of the tilt illusion and intra-hemispheric connectivity in V1 measured during perception of the tilt illusion, as opposed to intra-hemispheric connectivity measured during perception of an iso-oriented contextual stimulus, or intra-hemispheric connectivity in V2, V3, suggests the involvement of these orientation-dependent mechanisms. By contrast, the common increases in effective connectivity from non-contextual to contextual stimulation, observed in all early visual cortices (V1, V2, V3) and for both tilt illusion stimulus and iso-oriented contextual stimulus, suggest a generic, orientation-independent modulation exerted by contextual stimulation (Fig. 5).

In contrast to our findings, several fMRI and EEG studies show that neural activity as early as V1 is modulated by inputs from both contralateral and ipsilateral hemifields, which hint towards a joint contribution of intra- and inter-hemispheric integration to visual perception (Knyazeva et al., 1999, 2005, 2006; Ban et al., 2006; Stark and Milham, 2008). The discrepancy with the present findings may reflect the diversity and complexity of integration mechanisms involved in visual processing and visual perception. It would be of interest for future studies to test the contribution of intra- versus inter-hemispheric integration in other perceptual tasks, such as illusory contour perception and illusory size perception (Song et al. 2011, 2013b, 2017b; Harris et al., 2011). It is possible that our observation is specific to illusory orientation perception and results from the small receptive field sizes of neurons in V1. For perceptual illusions that involve higher visual areas where the receptive field sizes of neurons are larger and the integrations between neurons are stronger, inter-hemispheric integration may play a more important role.

Cases of split-brain patients, for whom the loss or surgical removal of callosal connections does not impair their ability to have visual experience within individual hemifields, already indicate that inter-hemispheric integration may not be as essential to visual perception as intra-hemispheric integration (Zaidel and Iacoboni, 2003). Now, we showed a similar lack of inter-hemispheric involvement in perception of bilateral stimuli in healthy participants. Together these observations touch upon an intriguing and longstanding question: why does the human brain have two hemispheres in the first place? Given the high wiring and metabolic costs of callosal connections, having a single hemisphere could be more space- and energy-efficient. So, why have two hemispheres? A two-hemisphere structure could be a consequence or coincidence arising from the bilateral symmetry of the human body. But above and beyond that, a two-hemisphere structure allows human conscious experience to have a certain degree of degeneracy, where the two hemispheres can each independently support conscious experience and consciousness does not depend on having effective connectivity between hemispheres (Tononi et al. 1999, 2016; Zaidel and Iacoboni, 2003). This is to be contrasted with the case of neocortical arealization, where the cortex is divided into areas of different functions and a lack of effective connectivity between these areas often leads to loss of consciousness, as evident in sleep and coma (Massimini et al., 2005; Rosanova and Massimini, 2012). Possibly, the design of two hemispheres and the design of neocortical arealization are complementary, ensuring both degeneracy and efficiency in the human brain.
